# *CDKAL1* rs7756992 is associated with diabetic retinopathy in a Chinese population with type 2 diabetes

**DOI:** 10.1038/s41598-017-09010-w

**Published:** 2017-08-18

**Authors:** Danfeng Peng, Jie Wang, Rong Zhang, Feng Jiang, Claudia H. T. Tam, Guozhi Jiang, Tao Wang, Miao Chen, Jing Yan, Shiyun Wang, Dandan Yan, Zhen He, Ronald C. W. Ma, Yuqian Bao, Cheng Hu, Weiping Jia

**Affiliations:** 1 Shanghai Diabetes Institute, Shanghai Key Laboratory of Diabetes Mellitus, Shanghai Clinical Centre for Diabetes, Shanghai Key Clinic Centre for Metabolic Diseases, Department of Endocrinology and Metabolism, Shanghai Jiao Tong University Affiliated Sixth People’s Hospital, Shanghai, China; 2 Department of Medicine and Therapeutics, The Chinese University of Hong Kong, Hong Kong, China; 3 Shanghai Jiao Tong University Affiliated Sixth People’s Hospital South Campus, Shanghai, China

## Abstract

Diabetic retinopathy (DR) is a major microvascular complication of diabetes. Susceptibility genes for type 2 diabetes may also impact the susceptibility of DR. This case-control study investigated the effects of 88 type 2 diabetes susceptibility loci on DR in a Chinese population with type 2 diabetes performed in two stages. In stage 1, 88 SNPs were genotyped in 1,251 patients with type 2 diabetes, and we found that *ADAMTS9-AS2* rs4607103, *WFS1* rs10010131, *CDKAL1* rs7756992, *VPS26A* rs1802295 and *IDE-KIF11-HHEX* rs1111875 were significantly associated with DR. The association between *CDKAL1* rs7756992 and DR remained significant after Bonferroni correction for multiple comparisons (corrected *P* = 0.0492). Then, the effect of rs7756992 on DR were analysed in two independent cohorts for replication in stage 2. Cohort (1) consisted of 380 patients with DR and 613 patients with diabetes for ≥5 years but without DR. Cohort (2) consisted of 545 patients with DR and 929 patients with diabetes for ≥5 years but without DR. A meta-analysis combining the results of stage 1 and 2 revealed a significant association between rs7756992 and DR, with the minor allele A conferring a lower risk of DR (OR 0.824, 95% CI 0.743–0.914, *P* = 2.46 × 10^−4^).

## Introduction

Chronic complications are the major causes of morbidity and mortality for patients with type 2 diabetes. As one of the most common chronic microvascular complications, diabetic retinopathy (DR) is a leading cause of blindness in working-age adults globally^1^. The overall prevalence of DR is 35.4% and is higher in patients with type 1 diabetes compared to those with type 2 diabetes (77.3% vs 25.2%)^2^. The prevalence of DR among patients with diabetes in China varies from 11.9% to 43.1%^3–5^. Given that China has the most patients suffering from diabetes around the world^6^, the number of patients with DR could be quite large, highlighting what is certainly a huge public health and financial burden for the country.

The aetiology of DR is complex and remains to be fully elucidated. Large-scale prospective studies have shown that the durations of diabetes, hyperglycaemia and hypertension are the most clinically important risk factors for DR^7, 8^. Furthermore, multiple studies have suggested that genetic factors also play important roles in the development of DR with an estimated heritability of 25% for DR and 50% for proliferative diabetic retinopathy (PDR)^9–11^. Therefore, the elucidation of genetic susceptibility factors is helpful for revealing the pathogenesis of DR. To date, several genome-wide association studies (GWAS) in populations of different ancestries have identified some potential susceptibility loci for DR^12, 13^. However, only one locus, rs9896052 near *GRB2*, showed an association that reached the genome-wide significance level for sight-threatening DR^13^, and few loci have been replicated in other studies^14, 15^. Numerous studies have attempted to identify susceptibility loci for DR through a candidate gene approach. Multiple genes, such as *VEGF*, *PPARG*, *EPO*, *AKR1B1*, *PPKCB*, *ACE* and *ICAM-1*, have been suggested to be associated with DR^14^. However, few of these studies have been consistently replicated^14, 16, 17^. Recently, a new approach, whole exome sequencing, have been applied for the identification for potential susceptibility loci, and some new candidate genes for DR or PDR were reported^18, 19^.

As a complex genetic disorder, about 90 susceptibility loci have been identified for type 2 diabetes through GWAS to date^20^. Recently, a study based the Singapore Epidemiology of Eye Diseases Study indicated that participants with more type 2 diabetes genetic risk alleles had higher risk of DR^21^. There are also several studies that investigated the association between type 2 diabetes susceptibility genes and DR. *TCF7L2* rs7903146 was reported to be associated with PDR in Caucasian patients with type 2 diabetes^22^, and *KCNJ11* rs5219 was reported to be associated with DR in a Chinese population with type 2 diabetes^23^. However, most of those studies included only one locus or a few loci, and the sample size was relative small. Associations between most of the type 2 diabetes susceptibility loci and DR have not been investigated, especially in Chinese population. So, we performed the present study to investigate the effects of over 80 type 2 diabetes susceptibility loci on DR in a Chinese population with type 2 diabetes.

## Results

### Associations between SNPs and DR in stage 1

We first analysed the effects of these SNPs on DR in stage 1 samples. DR and diabetic kidney disease (DKD) are two important microvascular diabetes complications with a high concordance rate in patients with diabetes, and DR and DKD might share common pathogenesis. Because of the close relationship between DKD and DR, DKD could be a considerably important confounding factor when we conduct the genetic association analysis for DR. To minimize the confounding influence of DKD on the effects of SNPs on DR, we divided the participants into four groups. These four groups formed two small case-control studies for DR according to the status of DKD: patients with DR only vs control patients without DR or DKD, patients with both DR and DKD vs patients with DKD only. In both case-control studies, association between SNPs and DR was examined. A meta-analysis was done to combine the results. The distributions of SNPs among these four groups were shown in Supplementary Table 1. As shown in Table 1, with adjustment for diabetes duration, HbA_1c_, blood pressure and body mass index (BMI), five loci (*ADAMTS9-AS2* rs4607103, *WFS1* rs10010131, *CDKAL1* rs7756992, *VPS26A* rs1802295 and *IDE-KIF11-HHEX* rs1111875) were significantly associated with DR, with rs7756992 showing the strongest association (OR 0.746, 95% CI 0.608–0.915, *P* = 0.0048 for the rs4607103 T allele; OR 1.629, 95% CI 1.019–2.606, *P* = 0.0416 for the rs10010131 A allele; OR 0.703, 95% CI 0.580–0.851, *P* = 0.0003 for the rs7756992 A allele; OR 0.673, 95% CI 0.483–0.939, P = 0.0197 for the rs1802295 T allele; and OR 0.808, 95% CI 0.656–0.995, *P* = 0.0448 for the rs1111875 C allele). However, on the basis of 164 independent tests, only the association between *CDKAL1* rs7756992 and DR remained significant after Bonferroni correction for multiple comparisons (corrected *P* = 0.0492 for rs7756992).

**Table 1:** Effects of the SNPs on DR in stage 1 samples.

Chr.	SNP	Position (Build 38)	Gene	Minor/major allele	Risk allele	Controls vs DR only		DKD only vs DKD&DR		Meta-analysis	
						OR (95% CI)	*P* ^*a*^	OR (95% CI)	*P* ^*a*^	OR (95% CI)	*P* ^*a*^
1	rs2641348	119895261	*ADAM30*	C/T	C	1.200 (0.586, 2.455)	0.62	1.020 (0.492, 2.116)	0.96	1.108 (0.665, 1.847)	0.69
1	rs10923931	119975336	*NOTCH2*	T/G	T	1.132 (0.562, 2.283)	0.73	0.918 (0.438, 1.924)	0.82	1.025 (0.616, 1.705)	0.92
1	rs340874	213985913	*PROX1*	G/A	G	1.162 (0.878, 1.540)	0.29	0.946 (0.726, 1.232)	0.68	1.042 (0.859, 1.263)	0.68
2	rs7578597	43505684	*THADA*	C/T	C	1.060 (0.162, 6.924)	0.95	0.971 (0.260, 3.625)	0.97	1.000 (0.340, 2.938)	1.00
2	rs243021	60357684	*LOC105374756*	C/T	T	0.978 (0.722, 1.324)	0.89	1.009 (0.766, 1.329)	0.95	0.995 (0.811, 1.220)	0.96
2	rs7593730	160314943	*RBMS1*	T/C	C	0.795 (0.538, 1.173)	0.25	0.909 (0.640, 1.292)	0.60	0.856 (0.659, 1.111)	0.24
2	rs3923113	164645339	*GRB14*	G/T	G	0.921 (0.563, 1.506)	0.74	1.489 (1.026, 2.160)	**0.0361**	1.250 (0.929, 1.682)	0.14
2	rs13389219	164672366	*LOC101929615*	T/C	T	0.854 (0.504, 1.449)	0.56	1.316 (0.822, 2.107)	0.25	1.087 (0.765, 1.545)	0.64
2	rs16856187	168913876	*G6PC2*	C/A	A	0.941 (0.687, 1.290)	0.71	0.874 (0.646, 1.182)	0.38	0.906 (0.728, 1.126)	0.37
2	rs7578326	226155937	*LOC646736*	G/A	A	0.946 (0.645, 1.385)	0.77	0.810 (0.548, 1.197)	0.29	0.877 (0.667, 1.152)	0.34
2	rs2943641	226229029	*LOC646736*	T/C	T	1.146 (0.613, 2.146)	0.67	0.939 (0.546, 1.617)	0.82	1.023 (0.679, 1.542)	0.91
3	rs1801282	12351626	*PPARG*	G/C	G	0.921 (0.493, 1.719)	0.80	2.048 (1.085, 3.863)	**0.0269**	1.364 (0.874, 2.129)	0.17
3	rs7612463	23294959	*UBE2E2*	A/C	C	0.915 (0.648, 1.292)	0.61	0.937 (0.676, 1.300)	0.70	0.927 (0.731, 1.175)	0.53
3	rs831571	64062621	*PSMD6*	T/C	C	0.895 (0.659, 1.215)	0.48	1.038 (0.797, 1.352)	0.78	0.974 (0.798, 1.190)	0.80
3	rs4607103	64726228	*ADAMTS9-AS2*	T/C	C	0.719 (0.534, 0.967)	**0.0292**	0.771 (0.582, 1.021)	0.07	0.746 (0.608, 0.915)	**0.0048**
3	rs4402960	185793899	*IGF2BP2*	T/G	G	1.000 (0.728, 1.372)	1.00	0.927 (0.694, 1.237)	0.61	0.959 (0.775, 1.187)	0.70
3	rs7651090	185795604	*IGF2BP2*	G/A	A	1.060 (0.770, 1.461)	0.72	0.922 (0.687, 1.239)	0.59	0.983 (0.791, 1.221)	0.88
3	rs16861329	186948673	*ST64GAL1*	T/C	C	0.980 (0.688, 1.397)	0.91	0.964 (0.676, 1.373)	0.84	0.972 (0.757, 1.248)	0.82
4	rs6815464	1316113	*MAEA*	G/C	C	0.918 (0.683, 1.233)	0.57	1.046 (0.808, 1.355)	0.73	0.988 (0.814, 1.201)	0.91
4	rs10010131	6291188	*WFS1*	A/G	A	1.910 (0.930, 3.923)	0.08	1.448 (0.779, 2.692)	0.24	1.629 (1.019, 2.606)	**0.0416**
5	rs459193	56510924	*C5orf67*	T/C	C	0.899 (0.679, 1.192)	0.46	0.975 (0.745, 1.278)	0.86	0.938 (0.772, 1.140)	0.52
5	rs4457053	77129124	*ZBED3-AS1*	G/A	A	0.837 (0.460, 1.524)	0.56	1.087 (0.595, 1.986)	0.79	0.953 (0.623, 1.458)	0.82
6	rs7756992	20679478	*CDKAL1*	A/G	G	0.697 (0.522, 0.931)	**0.0145**	0.707 (0.547, 0.913)	**0.0079**	0.703 (0.580, 0.851)	**0.0003**
6	rs9470794	38139068	*ZFAND3*	C/T	C	1.174 (0.873, 1.577)	0.29	1.007 (0.765, 1.326)	0.96	1.081 (0.884, 1.322)	0.45
6	rs1535500	39316274	*KCNK16*	T/G	T	1.239 (0.936, 1.640)	0.13	1.075 (0.833, 1.386)	0.58	1.146 (0.949, 1.384)	0.16
7	rs2191349	15024684	*GTF3AP5*	G/T	G	1.261 (0.952, 1.671)	0.11	0.977 (0.750, 1.273)	0.87	1.101 (0.908, 1.335)	0.33
7	rs864745	28140937	*JAZF1*	G/A	G	1.209 (0.856, 1.709)	0.28	0.992 (0.714, 1.378)	0.96	1.090 (0.859, 1.383)	0.48
7	rs1799884	44189469	*GCK*	A/G	A	1.276 (0.920, 1.770)	0.15	1.034 (0.763, 1.400)	0.83	1.139 (0.912, 1.423)	0.25
7	rs917793	44206254	*YKT6*	A/T	A	1.190 (0.856, 1.654)	0.30	1.082 (0.800, 1.463)	0.61	1.130 (0.905, 1.412)	0.28
7	rs6467136	127524904	*LOC105375490*	A/G	G	0.847 (0.585, 1.224)	0.38	0.791 (0.559, 1.117)	0.18	0.816 (0.634, 1.050)	0.11
7	rs10229583	127606849	*PAX4*	A/G	G	0.958 (0.654, 1.404)	0.83	0.908 (0.639, 1.291)	0.59	0.931 (0.718, 1.206)	0.59
7	rs791595	128222749	*LOC101928423*	A/G	A	1.166 (0.800, 1.699)	0.43	1.270 (0.866, 1.863)	0.22	1.216 (0.930, 1.591)	0.15
7	rs972283	130782095	*LOC105375508*	A/G	G	1.077 (0.782, 1.483)	0.65	0.838 (0.630, 1.115)	0.23	0.937 (0.757, 1.159)	0.55
8	rs516946	41661730	*ANK1*	A/G	A	1.175 (0.731, 1.891)	0.51	1.302 (0.874, 1.941)	0.20	1.248 (0.919, 1.694)	0.16
8	rs515071	41661944	*ANK1*	T/C	T	1.347 (0.893, 2.033)	0.16	1.049 (0.732, 1.504)	0.79	1.169 (0.892, 1.533)	0.26
8	rs896854	94948283	*TP53INP1*	A/G	G	0.743 (0.546, 1.011)	0.06	0.926 (0.703, 1.219)	0.58	0.840 (0.684, 1.031)	0.10
8	rs13266634	117172544	*SLC30A8*	T/C	T	0.934 (0.698, 1.249)	0.65	1.250 (0.954, 1.637)	0.11	1.092 (0.896, 1.331)	0.38
9	rs7041847	4287466	*GLIS3*	A/G	G	1.199 (0.902, 1.595)	0.21	0.858 (0.661, 1.113)	0.25	0.999 (0.824, 1.211)	0.99
9	rs17584499	8879118	*PTPRD*	T/C	T	1.084 (0.674, 1.745)	0.74	1.013 (0.656, 1.565)	0.95	1.045 (0.758, 1.440)	0.79
9	rs10811661	22134095	*CDKN2A/B*	C/T	C	1.006 (0.754, 1.342)	0.97	1.217 (0.929, 1.594)	0.15	1.113 (0.914, 1.356)	0.29
9	rs13292136	79337213	*CHCHD2P9*	T/C	C	1.150 (0.671, 1.972)	0.61	0.794 (0.485, 1.300)	0.36	0.940 (0.653, 1.353)	0.74
9	rs2796441	81694033	*LOC101927502*	C/T	C	1.622 (1.209, 2.176)	**0.0013**	1.025 (0.794, 1.322)	0.85	1.282 (0.817, 2.009)	0.28
9	rs11787792	136357696	*GPSM1*	G/A	G	0.708 (0.355, 1.412)	0.33	1.662 (0.772, 3.579)	0.19	1.037 (0.621, 1.733)	0.89
10	rs10906115	12272998	*CDC123*	G/A	A	0.843 (0.624, 1.138)	0.26	0.874 (0.667, 1.145)	0.33	0.860 (0.704, 1.051)	0.14
10	rs12779790	12286011	*CDC123*	G/A	G	0.877 (0.612, 1.255)	0.47	1.163 (0.827, 1.635)	0.39	1.017 (0.794, 1.302)	0.89
10	rs1802295	69171718	*VPS26A*	T/C	C	0.842 (0.535, 1.324)	0.46	0.520 (0.319, 0.847)	**0.0086**	0.673 (0.483, 0.939)	**0.0197**
10	rs12571751	79182874	*ZMIZ1*	G/A	G	1.274 (0.962, 1.687)	0.09	0.943 (0.731, 1.217)	0.65	1.080 (0.894, 1.305)	0.42
10	rs1111875	92703125	*IDE-KIF11-HHEX*	C/T	T	0.720 (0.523, 0.991)	**0.0439**	0.880 (0.669, 1.157)	0.36	0.808 (0.656, 0.995)	**0.0448**
10	rs7903146	112998590	*TCF7L2*	T/C	C	0.820 (0.431, 1.563)	0.55	0.924 (0.469, 1.818)	0.82	0.868 (0.544, 1.384)	0.55
10	rs10886471	119389891	*GRK5*	T/C	C	0.766 (0.543, 1.080)	0.13	0.829 (0.605, 1.137)	0.24	0.800 (0.634, 1.009)	0.06
11	rs231362	2670241	*KCNQ1*	T/C	T	1.281 (0.767, 2.140)	0.34	0.964 (0.621, 1.496)	0.87	1.087 (0.779, 1.518)	0.62
11	rs2237892	2818521	*KCNQ1*	T/C	C	0.794 (0.575, 1.096)	0.16	0.975 (0.719, 1.322)	0.87	0.885 (0.709, 1.104)	0.28
11	rs5219	17388025	*KCNJ11*	T/C	C	0.936 (0.701, 1.249)	0.65	0.977 (0.740, 1.288)	0.87	0.957 (0.783, 1.168)	0.66
11	rs10751301	78983593	*TENM4*	C/G	G	0.936 (0.670, 1.308)	0.70	0.934 (0.675, 1.292)	0.68	0.935 (0.741, 1.180)	0.57
11	rs1387153	92940662	*MTNR1B*	T/C	T	1.057 (0.799, 1.398)	0.70	1.214 (0.929, 1.587)	0.15	1.136 (0.936, 1.379)	0.20
12	rs10842994	27812217	*LOC105369709*	T/C	C	1.210 (0.829, 1.765)	0.32	0.803 (0.568, 1.135)	0.21	0.968 (0.750, 1.250)	0.80
12	rs1531343	65781114	*RPSAP52*	C/G	G	0.802 (0.554, 1.163)	0.24	0.856 (0.591, 1.241)	0.41	0.829 (0.637, 1.078)	0.16
12	rs7961581	71269322	*LOC105369832*	C/T	T	1.389 (0.972, 1.984)	0.07	0.698 (0.500, 0.973)	**0.034**	0.981 (0.500, 1.927)	0.96
13	rs9552911	23290518	*SGCG*	A/G	G	0.925 (0.656, 1.305)	0.66	1.031 (0.763, 1.392)	0.84	0.984 (0.784, 1.234)	0.89
13	rs1359790	80143021	*LOC105370275*	T/C	T	0.984 (0.710, 1.364)	0.92	1.110 (0.831, 1.482)	0.48	1.053 (0.848, 1.307)	0.64
15	rs7403531	38530704	*RASGRP1*	T/C	T	1.211 (0.912, 1.607)	0.19	1.174 (0.893, 1.544)	0.25	1.192 (0.979, 1.451)	0.08
15	rs7172432	62104190	*NPM1P47*	G/A	G	1.074 (0.797, 1.449)	0.64	1.269 (0.956, 1.684)	0.10	1.173 (0.955, 1.441)	0.13
15	rs1436955	62112183	*NPM1P47*	A/G	A	0.929 (0.661, 1.304)	0.67	1.226 (0.903, 1.665)	0.19	1.082 (0.862, 1.359)	0.49
15	rs7178572	77454848	*HMG20A*	G/A	G	1.061 (0.793, 1.419)	0.69	1.020 (0.774, 1.344)	0.89	1.039 (0.851, 1.270)	0.71
15	rs7177055	77540420	*LOC101929457*	A/G	A	1.019 (0.758, 1.369)	0.90	1.024 (0.780, 1.344)	0.86	1.022 (0.836, 1.248)	0.83
15	rs11634397	80139880	*ZFAND6*	G/A	G	1.125 (0.685, 1.847)	0.64	1.552 (1.032, 2.333)	**0.0346**	1.363 (0.995, 1.868)	0.05
15	rs2028299	89831025	*AP3S2*	C/A	A	0.936 (0.667, 1.314)	0.70	0.925 (0.678, 1.262)	0.62	0.930 (0.740, 1.170)	0.54
15	rs8042680	90978107	*PRC1*	C/A	A	0.342 (0.074, 1.585)	0.17	1.265 (0.197, 8.137)	0.80	0.580 (0.178, 1.896)	0.37
16	rs8050136	53782363	*FTO*	A/C	C	1.113 (0.745, 1.662)	0.60	0.866 (0.599, 1.252)	0.45	0.972 (0.741, 1.274)	0.83
16	rs7202877	75213347	*CTRB1*	G/T	T	0.928 (0.652, 1.321)	0.68	0.897 (0.661, 1.218)	0.49	0.910 (0.723, 1.147)	0.43
16	rs17797882	79373021	*MAF*	T/C	T	1.036 (0.716, 1.499)	0.85	1.145 (0.836, 1.568)	0.40	1.098 (0.864, 1.395)	0.44
16	rs16955379	81455768	*CMIP*	T/C	C	0.947 (0.684, 1.311)	0.74	0.905 (0.662, 1.237)	0.53	0.925 (0.738, 1.159)	0.50
17	rs391300	2312964	*SRR*	A/G	A	0.893 (0.651, 1.225)	0.48	1.206 (0.910, 1.599)	0.19	1.056 (0.855, 1.303)	0.61
17	rs312457	7037074	*SLC16A13*	C/T	C	1.228 (0.810, 1.861)	0.33	1.001 (0.715, 1.403)	0.99	1.086 (0.835, 1.411)	0.54
17	rs13342232	7042621	*SLC16A11*	G/A	G	1.262 (0.828, 1.924)	0.28	1.202 (0.850, 1.701)	0.30	1.226 (0.938, 1.602)	0.14
17	rs4430796	37738049	*HNF1B*	G/A	A	0.855 (0.641, 1.141)	0.29	1.089 (0.836, 1.417)	0.53	0.976 (0.803, 1.185)	0.80
18	rs12970134	60217517	*LOC342784*	A/G	A	1.095 (0.782, 1.532)	0.60	1.301 (0.963, 1.757)	0.09	1.205 (0.963, 1.508)	0.10
19	rs10401969	19296909	*SUGP1*	C/T	C	1.064 (0.661, 1.713)	0.80	0.987 (0.641, 1.518)	0.95	1.021 (0.742, 1.405)	0.90
19	rs3786897	33402102	*PEPD*	G/A	G	1.094 (0.825, 1.452)	0.53	1.062 (0.817, 1.381)	0.65	1.077 (0.888, 1.305)	0.45
20	rs6017317	44318326	*FITM2*	G/T	G	0.873 (0.654, 1.164)	0.35	1.138 (0.879, 1.473)	0.33	1.011 (0.834, 1.225)	0.91
20	rs4812829	44360627	*HNF4A*	A/G	G	0.790 (0.595, 1.049)	0.10	0.903 (0.701, 1.162)	0.43	0.851 (0.705, 1.028)	0.09
X	rs5945326	153634467	*DUSP9*	G/A	G	1.123 (0.792, 1.593)	0.51	1.209 (0.877, 1.667)	0.25	1.169 (0.923, 1.480)	0.20

DKD = diabetic kidney disease; DR = diabetic retinopathy.

*P* values < 0.05 are shown in bold.

^a^Adjusted for duration of diabetes, HbA_1c_, systolic blood pressure, diastolic blood pressure, body mass index under an additive model.

The OR with 95% CI shown is for the minor allele.

### Validation of the effect of *CDKAL1* rs7756992 on DR in stage 2

To further validate the effect of *CDKAL1* rs7756992 on DR, we genotyped this SNP in two independent cohorts in stage 2. As shown in Table 2, in Cohort (1), with adjustment for diabetes duration, HbA_1c_, blood pressure and BMI, We found that rs7756992 showed similar trend as those in stage 1 samples for DR (OR 0.890, 95% CI 0.728–1.088, *P* = 0.26). In Cohort (2), rs7756992 showed a marginal association with DR (OR 0.874, 95% CI 0.749–1.020, *P* = 0.09) following adjustment for confounders. Then we conducted a meta-analysis with the fixed-effect model (*P*_for homogeneity_ = 0.23), rs7756992 was significantly associated with DR, with the minor allele A conferring a lower risk of DR (OR 0.824, 95% CI 0.743–0.914, *P* = 2.46 × 10^−4^).

**Table 2:** Association of *CDKAL1* rs7756992 with DR in Chinese patients with type 2 diabetes.

	n (case/control)	Minor allele frequency		OR (95% CI)	*P* ^a^
		Control subjects	Case subjects		
DR vs controls	313/238	0.475	0.428	0.697 (0.522, 0.931)	**0.0145**
DR and DKD vs DKD	281/419	0.499	0.414	0.707 (0.547, 0.913)	**0.0079**
Validation cohort (1)	380/613	0.453	0.43	0.890 (0.728–1.088)	0.26
Validation cohort (2)	545/929	0.470	0.439	0.874 (0.749–1.020)	0.09
Meta-analysis	1519/2199	0.471	0.43	0.824 (0.743–0.914)	**2.46** × **10** ^**−4**^

DKD = diabetic kidney disease; DR = diabetic retinopathy.

*P* values < 0.05 are shown in bold.

^a^Adjusted for duration of diabetes, HbA_1c_, systolic blood pressure, diastolic blood pressure, body mass index.

The OR with 95% CI shown is for the minor allele.

### Effects of SNPs on DR severity

Further, we tried to examine the effect of *CDKAL1* rs7756992 on the disease severity of DR. From our sample population, there were 2,199 patients without DR, 709 with mild NPDR, 396 with moderate NPDR, 267 with severe NPDR, 116 with PDR and 31 patients with DR that lacked a severity assessment. However, we did not find any associations between this SNP and the severity of DR (Supplementary Table 2).

## Discussion

In this study, we analysed the effects of 82 susceptibility loci for type 2 diabetes on DR in a Chinese population with type 2 diabetes. A total of 3,718 participants were recruited. On the basis of an estimated effect size of genetic loci for DR (~1.2), our samples had >90% power to detect a SNP effect with a minor allele frequency (MAF) of 0.25 and >80% power to detect a SNP effect with a MAF of 0.15 at a level of significance of 0.05. With a two-stage design, we found that *CDKAL1* rs7756992 were significantly associated with DR (OR 0.824, *P* = 2.47 × 10^−4^), and the association between rs7756992 and DR remained significant after Bonferroni correction. To our knowledge, this study is the first to identify an association between this SNP and DR. Besides, we found another four loci that showed association with DR in stage 1. Although, the association between these loci and DR could not survive after Bonferroni correction, these loci might be potential candidate genes for DR and could be further investigated.

Numerous genetic studies have found that several SNPs within the *CDKAL1* region are associated with type 2 diabetes among multiple ethnic populations^24^. rs7756992 was one of the most commonly reported SNPs in *CDKAL1*, with the major G allele conferring a higher risk of type 2 diabetes^24^. It has also been reported to be associated with impaired insulin secretion^25, 26^. In this study, we found that rs7756992 was associated with DR, with the minor A allele conferring a lower risk of DR. So the major G allele was the risk allele for DR too. Liu *et al*.^27^ reported that another SNP in *CDKAL1*, rs10946398 which was also reported to be associated with type 2 diabetes in multiple populations^24^, was associated with DR in 580 Chinese patients with type 2 diabetes. Mice with a beta cell-specific knockout of *Cdkal1* presented decreased insulin secretion and impaired blood glucose control^28^. However, although some study indicated that rs7756992 was correlated with CDKAL1 protein level, the underlying mechanism has not yet been elucidated^29^. And functional study which investigates the role of CDKAL1 in the pathophysiological process of retinopathy has not been reported yet. Thus, how *CDKAL1* rs7756992 impact the susceptibility of DR and whether it’s the causal locus of DR or not requires further investigation.

This study has some limitations. First, because the participants of this study were recruited from Shanghai and nearby regions, our findings may be specific to Chinese patients and may have inherent bias. This may explain why we did not find association between *TCF7L2* rs7903146, *KCNJ11* rs5219 and DR in this study. Second, lifestyle factors such as cigarette smoking and alcohol consumption were not included in the genotype-disease analysis. Whether interactions exist between lifestyle factors and these genetic variants in terms of DR remains unknown. Third, rs7756992 which was associated with DR in this study is located in the intron of *CDKAL1*. Thus, the relationships between rs7756992 and genes and how they modulate DR risk are largely unknown. Hence, studies in other ethnic populations are needed to further replicate our findings, and causal loci and genes should be identified to elucidate the underlying mechanism.

In summary, we identified *CDKAL1* rs7756992 as a susceptibility locus for DR in a Chinese population with type 2 diabetes. Further studies are needed to replicate this finding and to elucidate the underlying mechanism.

## Methods

### Participants

A two-stage approach was applied for this study. In stage 1, we recruited 1,251 patients with type 2 diabetes from the Shanghai Diabetes Institute Inpatient Database of Shanghai Jiao Tong University Affiliated Sixth People’s Hospital^15, 30^. These patients included 313 patients with DR but no DKD, 419 patients with DKD but no DR, 281 patients with both DR and DKD, and 238 control subjects with diabetes for ≥10 years but without DR or DKD. In stage 2, two independent cohorts were recruited for replication analysis. Cohort (1) recruited a total of 993 patients with type 2 diabetes from the Shanghai Diabetic Complications Study and Shanghai Diabetes Institute Inpatient Database^15, 31^, and consisted of 380 patients with DR and 613 patients with diabetes for ≥5 years but without DR. Cohort (2) recruited a total of 1,474 patients with type 2 diabetes from the Shanghai Diabetes Institute Inpatient Database, including 545 patients with DR and 929 patients with diabetes for ≥5 years but without DR. The basic characteristics of the participants are shown in Tables 3 and 4.

**Table 3:** Clinical characteristics of the participants in stage 1.

	Case-control study (1)		Case-control study (2)	
	Controls	DR only	DKD only	DR&DKD
Samples (n)	238	313	419	281
Male/female (n)	88/150	136/177	226/193	145/136
Age (years)	65.75 ± 9.39	59.8 ± 10.38	62.84 ± 13.77	65.4 ± 10.63
Diabetes duration (years)	12.4 (10, 15.91)	8.94 (4, 13)	6 (1, 10)	12 (8, 18)
BMI (kg/m^2^)	23.75 ± 3.12	23.95 ± 3.46	25.3 ± 3.67	24.29 ± 3.75
SBP (mmHg)	133.47 ± 16.77	134.19 ± 17.21	137.37 ± 18.4	143.85 ± 20.12
DBP (mmHg)	78.42 ± 8.52	80.04 ± 9.33	81.78 ± 10.01	82.77 ± 9.67
HbA_1c_ (%)	8.45 ± 1.93	9.01 ± 2.11	9.16 ± 2.34	9.36 ± 2.25
HbA_1c_ (mmol/mol)	68.79 ± 21.08	75.01 ± 23.02	76.60 ± 25.60	78.77 ± 24.58
AERs (mg/24 h)	8.44 (5.99, 12.53)	10.60 (7.17, 15.89)	67.57 (35.96, 159.43)	88.88 (37.04, 377.93)
eGFRs	122.38 (107.85, 141.29)	129.88 (113.48, 152.14)	106.32 (80.74, 134.58)	92.25 (71.17, 126.85)

Data are n, mean ± SD, or median (interquartile range).

AERs = albumin excretion rates; BMI = body mass index; DBP = diastolic blood pressure; DKD = diabetic kidney disease; DR = diabetic retinopathy; eGFR = estimated glomerular filtration rate; HbA_1c_ = haemoglobin A_1c_; SBP = systolic blood pressure.

**Table 4:** Clinical characteristics of the participants in stage 2.

	Cohort (1)		Cohort (2)	
	Controls	DR	Controls	DR
Samples (n)	613	380	929	545
Male/female (n)	289/324	188/192	529/400	303/242
Age (years)	63.69 ± 10.34	60.19 ± 11.23	59.74 ± 8.79	57.73 ± 9.24
Diabetes duration (years)	10 (7, 14)	10 (6, 15)	10 (7, 13)	11 (8, 16)
BMI (kg/m^2^)	24.74 ± 3.43	25.36 ± 3.65	24.86 ± 3.56	24.85 ± 3.5
SBP (mmHg)	133.59 ± 16.44	136.08 ± 19.29	130.99 ± 16.17	135.25 ± 17.68
DBP (mmHg)	80.83 ± 9.2	81.33 ± 9.82	79.99 ± 9.16	80.41 ± 9.29
HbA_1c_ (%)	8.05 ± 1.79	8.89 ± 2.34	8.44 ± 1.83	9.11 ± 2.1
HbA_1c_ (mmol/mol)	64.49 ± 19.59	73.61 ± 25.56	68.72 ± 19.96	76.09 ± 22.97
AERs (mg/24 h)	10.85 (6.41, 25.17)	17.26 (7.91, 71.16)	10.44 (6.65, 24.32)	17.25 (7.89, 84.24)
eGFRs	121.41 (103.49, 143.88)	123.98 (102.11, 148.34)	124.84 (105.89, 149.06)	130.66 (102.93, 154.61)

Data are n, mean ± SD, or median (interquartile range).

AERs = albumin excretion rates; BMI = body mass index; DBP = diastolic blood pressure; DKD = diabetic kidney disease; DR = diabetic retinopathy; eGFR = estimated glomerular filtration rate; HbA_1c_ = haemoglobin A_1c_; SBP = systolic blood pressure.

This study was approved by the Institutional Review Board of Shanghai Jiao Tong University Affiliated Sixth People’s Hospital. All experiments were performed in accordance with relevant guidelines and regulations. Written informed consent was obtained from each participant.

### Clinical assessment

A diagnosis of type 2 diabetes was based on the World Health Organization guidelines (1999)^32^. DR was diagnosed by fundus photography or a history of panretinal photocoagulation (scatter laser) treatment. Fundus photography was performed with a 45-degree 6.3-megapixel digital nonmydriatic camera (Canon CR6-45NM, Lake Success, NY, USA) according to a standardised protocol at the Department of Ophthalmology, Shanghai Jiao Tong University Affiliated Sixth People’s Hospital. Retinopathy was graded according to the International Classification of Diabetic Retinopathy as follows: mild non-proliferative DR (NPDR), moderate NPDR, severe NPDR, or PDR^33^. For each patient, both eyes were examined, and the more severely affected eye was used to classify the DR. For the definition of DR, a subject with any DR was considered as a DR case. The 24-h albumin excretion rate (AER) and estimated glomerular filtration rate (eGFR) were applied to assess DKD. The AER was measured over 3 consecutive days, and the mean value was recorded for each patient. eGFR was calculated using a formula developed by the Modification of Diet in Renal Disease study group with adjustment for Chinese ethnicity: 186 × (serum creatinine in mmol/L × 0.0113) – 1.154 × (age in years) – 0.203 × (0.742 if female) × (1.233 if Chinese)^34^. Patients with AER ≥ 30 mg/24 h or eGFR < 90 mL/min/1.73 m^2^ were diagnosed with DKD. Besides, patients with history of other renal diseases were excluded in the enrollment of study participants. Glycaemic control was evaluated by measuring haemoglobin A_1c_ (HbA_1c_) levels. Anthropometric parameters, blood pressure and lipid profile data were also collected for each participant.

### Single-nucleotide polymorphism (SNP) selection and genotyping

In stage 1, 88 SNPs which were previously reported to be associated with type 2 diabetes by GWAS or large-scale association studies were genotyped, including rs340874, rs780094, rs243021, rs998451, rs7593730, rs3923113, rs13389219, rs16856187, rs7578326, rs2943641, rs7612463, rs831571, rs11708067, rs4402960, rs16861329, rs6815464, rs459193, rs4457053, rs9470794, rs1535500, rs2191349, rs1799884, rs917793, rs6467136, rs10229583, rs791595, rs972283, rs516946, rs515071, rs896854, rs7041847, rs17584499, rs13292136, rs2796441, rs11787792, rs10906115, rs1802295, rs12571751, rs10886471, rs231362, rs2237892, rs1552224, rs10751301, rs1387153, rs10842994, rs1531343, rs7957197, rs9552911, rs1359790, rs7403531, rs7172432, rs1436955, rs7178572, rs7177055, rs11634397, rs2028299, rs8042680, rs7202877, rs17797882, rs16955379, rs391300, rs312457, rs13342232, rs4430796, rs12970134, rs10401969, rs3786897, rs6017317, rs4812829, rs5945326, rs864745, rs10811661, rs2641348, rs7578597, rs1801282, rs4607103, rs7651090, rs10010131, rs7756992, rs10946398, rs13266634, rs12779790, rs1111875, rs7903146, rs5219, rs7961581, rs8050136, and rs10923931^20, 35^. The SNPs that showed associations with DR were genotyped in stage 2 samples. All of the SNPs were genotyped using a MassARRAY iPLEX system (MassARRAY Compact Analyser, Sequenom, San Diego, CA, USA). The genotyping data underwent a series of quality control checks. The concordance rate based on 100 duplicates was greater than 99% for all SNPs. Sample call rate was greater than 85% for all samples. The Hardy–Weinberg equilibrium test was performed before the statistical analysis (a two-tailed *P* value < 0.01 was considered statistically significant), and rs1552224 and rs10946398 were excluded. rs780094 was exclude due to call rate <90%. Another 3 SNPs (rs7957197, rs998451 and rs11708067) were rare in our population (minor allele frequency < 0.0015) and were excluded from statistical analyses.

### Statistical analysis

Logistic regression was used to compare the difference in genotype distributions between patients with or without DR under an additive model with adjustment for confounders using PLINK (v1.07)^36^; odds ratios (ORs) with 95% confidence intervals (CIs) are presented with reference to the minor allele. Combined ORs from different studies were calculated using a Comprehensive Meta-Analysis (v2.2.057) with a fixed- or random-effect model after testing for heterogeneity. The test for homogeneity was assessed with the Cochran Q test. The effects of SNPs on the level of DR severity were analysed by trend analysis using SAS 9.3 (SAS Institute, Cary, NC, USA). Bonferroni correction was applied to adjust for multiple comparisons. Statistical significance was defined as a two-tailed *P* value < 0.05.

## Electronic supplementary material

supplementary tables
