# Supplementary material for: CDKAL1 rs7756992 is associated with diabetic retinopathy in a Chinese population with type 2 diabetes
Source: Sci Rep. 2017 Aug 18;7:8812. doi: 10.1038/s41598-017-09010-w (PMC5562862; doi:10.1038/s41598-017-09010-w)
Supplement: Supplementary file 1 — supplementary tables [file 41598_2017_9010_MOESM1_ESM.pdf]

CDKAL1 rs7756992 is associated with diabetic retinopathy in a Chinese population with type 2 diabetes

Danfeng Peng[1+], Jie Wang[1+], Rong Zhang[1], Feng Jiang[1], Claudia HT Tam[2], Guozhi Jiang[2], Tao Wang[1], Miao Chen[1], Jing Yan[1], Shiyun Wang[1], Dandan Yan[1], Zhen He[1], Ronald CW Ma[2], Yuqian Bao[1], Cheng Hu[1,3*] & Weiping Jia[1*]

Supplementary Table 1 Distributions of SNPs among stage 1 samples

Chr.	SNP	Gene	Minor/major allele	Minor allele frequency			
				Case-control study (1)		Case-control study (2)	
				Controls	DR only	DN only	DR&DN
1	rs2641348	*ADAM30*	C/T	0.038	0.040	0.033	0.031
1	rs10923931	*NOTCH2*	T/G	0.040	0.040	0.034	0.032
1	rs340874	*PROX1*	G/A	0.393	0.440	0.378	0.377
2	rs7578597	*THADA*	C/T	0.007	0.005	0.006	0.008
2	rs243021	*LOC105374756*	C/T	0.328	0.315	0.312	0.310
2	rs7593730	*RBMS1*	T/C	0.145	0.133	0.151	0.152
2	rs3923113	*GRB14*	G/T	0.110	0.094	0.108	0.144
2	rs13389219	*LOC101929615*	T/C	0.079	0.078	0.081	0.097
2	rs16856187	*G6PC2*	C/A	0.274	0.242	0.282	0.252
2	rs7578326	*LOC646736*	G/A	0.147	0.138	0.158	0.131
2	rs2943641	*LOC646736*	T/C	0.051	0.072	0.053	0.065
3	rs1801282	*PPARG*	G/C	0.045	0.054	0.032	0.059
3	rs7612463	*UBE2E2*	A/C	0.195	0.196	0.200	0.185
3	rs831571	*PSMD6*	T/C	0.359	0.351	0.356	0.370
3	rs4607103	*ADAMTS9-AS2*	T/C	0.418	0.354	0.383	0.358
3	rs4402960	*IGF2BP2*	T/G	0.258	0.278	0.278	0.262
3	rs7651090	*IGF2BP2*	G/A	0.258	0.283	0.283	0.261
3	rs16861329	*ST64GAL1*	T/C	0.186	0.198	0.170	0.167
4	rs6815464	*MAEA*	G/C	0.421	0.413	0.390	0.381
4	rs10010131	*WFS1*	A/G	0.037	0.062	0.043	0.051
5	rs459193	*C5orf67*	T/C	0.426	0.409	0.438	0.451
5	rs4457053	*ZBED3-AS1*	G/A	0.053	0.051	0.049	0.048
6	rs7756992	*CDKAL1*	A/G	0.475	0.428	0.499	0.414
6	rs9470794	*ZFAND3*	C/T	0.306	0.328	0.314	0.340
6	rs1535500	*KCNK16*	T/G	0.434	0.466	0.459	0.495
7	rs2191349	*GTF3AP5*	G/T	0.316	0.355	0.350	0.348
7	rs864745	*JAZF1*	G/A	0.208	0.204	0.223	0.224
7	rs1799884	*GCK*	A/G	0.223	0.280	0.225	0.224

Supplementary Table 1 (Continued) Distributions of SNPs among stage 1 samples

Chr.	SNP	Gene	Minor/major allele	Minor allele frequency			
				Case-control study (1)		Case-control study (2)	
				Controls	DR only	DN only	DR&DN
7	rs917793	*YKT6*	A/T	0.227	0.271	0.218	0.220
7	rs6467136	*LOC105375490*	A/G	0.183	0.175	0.203	0.165
7	rs10229583	*PAX4*	A/G	0.138	0.152	0.173	0.167
7	rs791595	*LOC101928423*	A/G	0.156	0.163	0.132	0.158
7	rs972283	*LOC105375508*	A/G	0.246	0.257	0.295	0.265
8	rs516946	*ANK1*	A/G	0.084	0.109	0.101	0.112
8	rs515071	*ANK1*	T/C	0.122	0.163	0.150	0.143
8	rs896854	*TP53INP1*	A/G	0.338	0.307	0.314	0.306
8	rs13266634	*SLC30A8*	T/C	0.354	0.352	0.374	0.411
9	rs7041847	*GLIS3*	A/G	0.434	0.486	0.462	0.438
9	rs17584499	*PTPRD*	T/C	0.091	0.098	0.095	0.100
9	rs10811661	*CDKN2A/B*	C/T	0.370	0.385	0.410	0.409
9	rs13292136	*CHCHD2P9*	T/C	0.070	0.075	0.094	0.082
9	rs2796441	*LOC101927502*	C/T	0.369	0.458	0.405	0.411
9	rs11787792	*GPSM1*	G/A	0.049	0.034	0.029	0.032
10	rs10906115	*CDC123*	G/A	0.365	0.356	0.352	0.316
10	rs12779790	*CDC123*	G/A	0.190	0.174	0.183	0.174
10	rs1802295	*VPS26A*	T/C	0.110	0.111	0.099	0.078
10	rs12571751	*ZMIZ1*	G/A	0.410	0.463	0.463	0.436
10	rs1111875	*IDE-KIF11-HHEX*	C/T	0.314	0.290	0.301	0.289
10	rs7903146	*TCF7L2*	T/C	0.041	0.040	0.041	0.047
10	rs10886471	*GRK5*	T/C	0.217	0.198	0.226	0.185
11	rs231362	*KCNQ1*	T/C	0.070	0.101	0.100	0.093
11	rs2237892	*KCNQ1*	T/C	0.262	0.255	0.284	0.270
11	rs5219	*KCNJ11*	T/C	0.434	0.414	0.426	0.408
11	rs10751301	*TENM4*	C/G	0.230	0.209	0.204	0.209
11	rs1387153	*MTNR1B*	T/C	0.441	0.439	0.426	0.441
12	rs10842994	*LOC105369709*	T/C	0.181	0.189	0.188	0.161
12	rs1531343	*RPSAP52*	C/G	0.164	0.136	0.137	0.128
12	rs7961581	*LOC105369832*	C/T	0.175	0.245	0.245	0.203
13	rs9552911	*SGCG*	A/G	0.240	0.212	0.234	0.244
13	rs1359790	*LOC105370275*	T/C	0.252	0.241	0.273	0.270
15	rs7403531	*RASGRP1*	T/C	0.366	0.400	0.358	0.418
15	rs7172432	*NPM1P47*	G/A	0.341	0.363	0.361	0.394
15	rs1436955	*NPM1P47*	A/G	0.217	0.201	0.216	0.244
15	rs7178572	*HMG20A*	G/A	0.376	0.384	0.356	0.357
15	rs7177055	*LOC101929457*	A/G	0.351	0.352	0.349	0.339
15	rs11634397	*ZFAND6*	G/A	0.083	0.095	0.093	0.129
15	rs2028299	*AP3S2*	C/A	0.212	0.202	0.214	0.198

Supplementary Table 1 (Continued) Distributions of SNPs among stage 1 samples

Chr.	SNP	Gene	Minor/major allele	Minor allele frequency			
				Case-control study (1)		Case-control study (2)	
				Controls	DR only	DN only	DR&DN
15	rs8042680	*PRC1*	C/A	0.011	0.005	0.005	0.004
16	rs8050136	*FTO*	A/C	0.126	0.141	0.156	0.148
16	rs7202877	*CTRB1*	G/T	0.206	0.184	0.219	0.214
16	rs17797882	*MAF*	T/C	0.194	0.218	0.202	0.222
16	rs16955379	*CMIP*	T/C	0.267	0.257	0.239	0.235
17	rs391300	*SRR*	A/G	0.284	0.283	0.326	0.341
17	rs312457	*SLC16A13*	C/T	0.120	0.152	0.159	0.161
17	rs13342232	*SLC16A11*	G/A	0.117	0.147	0.140	0.158
17	rs4430796	*HNF1B*	G/A	0.393	0.349	0.296	0.300
18	rs12970134	*LOC342784*	A/G	0.218	0.220	0.212	0.251
19	rs10401969	*SUGP1*	C/T	0.080	0.089	0.093	0.090
19	rs3786897	*PEPD*	G/A	0.415	0.450	0.458	0.455
20	rs6017317	*FITM2*	G/T	0.494	0.477	0.416	0.476
20	rs4812829	*HNF4A*	A/G	0.487	0.458	0.468	0.450
X	rs5945326	*DUSP9*	G/A	0.308	0.350	0.355	0.398

Supplementary Table 2 Distribution of rs7756992 among patients with different severities of diabetic retinopathy

	n	Genotype count 11/12/22[a]	Minor allele frequncy	*P* value for trend analysis
Patients without DR	2,199	608/989/488	0.471	
Mild NPDR	709	242/323/129	0.419	
Moderate NPDR	396	126/174/79	0.438	
Severe NPDR	267	79/117/55	0.452	
PDR	116	38/53/20	0.419	0.96

[a] 11, major allele homozygotes; 12, heterozygotes; 22, minor allele homozygotes

DR = diabetic retinopathy; NPDR = non-proliferative diabetic retinopathy; PDR = proliferative diabetic retinopathy.
